# Impact of performance and information feedback on medical interns' confidence–accuracy calibration

**DOI:** 10.1007/s10459-023-10252-9

**Published:** 2023-06-17

**Authors:** J. Staal, K. Katarya, M. Speelman, R. Brand, J. Alsma, J. Sloane, W. W. Van den Broek, L. Zwaan

**Affiliations:** 1 Institute of Medical Education Research, Erasmus University Medical Center Rotterdam, Rotterdam, The Netherlands; 2 Faculty of Medical Sciences, Erasmus University Medical Center Rotterdam, Rotterdam, The Netherlands; 3 Department of Internal Medicine, Franciscus Gasthuis & Vlietland, Rotterdam, The Netherlands; 4 Intensive Care Unit, Haaglanden Medical Center Den Haag, The Hague, The Netherlands; 5 Department of Internal Medicine, Erasmus University Medical Center Rotterdam, Rotterdam, The Netherlands; 6 Center for Innovations in Quality, Effectiveness and Safety, Michael E. DeBakey VA Medical Center and Department of Medicine, Baylor College of Medicine, Houston, TX USA

**Keywords:** Calibration, Clinical reasoning, Diagnostic error, Feedback, Medical education

## Abstract

Diagnostic errors are a major, largely preventable, patient safety concern. Error interventions cannot feasibly be implemented for every patient that is seen. To identify cases at high risk of error, clinicians should have a good calibration between their perceived and actual accuracy. This experiment studied the impact of feedback on medical interns’ calibration and diagnostic process. In a two-phase experiment, 125 medical interns from Dutch University Medical Centers were randomized to receive no feedback (control), feedback on their accuracy (performance feedback), or feedback with additional information on why a certain diagnosis was correct (information feedback) on 20 chest X-rays they diagnosed in a feedback phase. A test phase immediately followed this phase and had all interns diagnose an additional 10 X-rays without feedback. Outcome measures were confidence–accuracy calibration, diagnostic accuracy, confidence, and time to diagnose. Both feedback types improved overall confidence–accuracy calibration (R^2^_No Feedback_ = 0.05, R^2^_Performance Feedback_ = 0.12, R^2^_Information Feedback_ = 0.19), in line with the individual improvements in diagnostic accuracy and confidence. We also report secondary analyses to examine how case difficulty affected calibration. Time to diagnose did not differ between conditions. Feedback improved interns’ calibration. However, it is unclear whether this improvement reflects better confidence estimates or an improvement in accuracy. Future research should examine more experienced participants and non-visual specialties. Our results suggest that feedback is an effective intervention that could be beneficial as a tool to improve calibration, especially in cases that are not too difficult for learners.

## Introduction

Diagnostic errors are defined as missed, delayed, or wrong diagnoses and form a threat to achieving high quality care (National Academies of Sciences, Engineering, and Medicine, 2015). It is estimated that in the United States alone, 12 million adults are affected by diagnostic errors yearly (Singh et al., 2014), even though 80% are estimated to be preventable (Zwaan et al., 2010). Moreover, diagnostic errors resulted in higher mortality rates when compared with other adverse events (i.e., errors that resulted in unintended harm) (Zwaan et al., 2010). Given the major implications for patient safety, it is crucial to develop strategies to prevent diagnostic errors.

Research shows that diagnostic errors are primarily caused by flaws in clinician’s cognitive processes, often in combination with technical and organizational factors (Singh & Zwaan, 2016). One often proposed strategy that could improve such cognitive errors on an individual level is feedback. For example, feedback provided via clinical audits has been shown effective in improving the quality of professional practice and adherence to guidelines (Jamtvedt et al., 2006). Feedback is also often recommended to improve the clinical reasoning processes of individual clinicians and reduce potential cognitive flaws (Berner & Graber, 2008; Croskerry, 2000; Meyer & Singh, 2019; Zwaan & Hautz, 2019).

The mechanism underlying the assumption that feedback might improve clinicians’ reasoning processes is often referred to as calibration (Croskerry, 2000; Meyer & Singh, 2019). This concept defines the alignment between clinicians’ confidence in their accuracy and their actual diagnostic accuracy. When clinicians are well-calibrated, they can make an accurate assessment of their own performance and will be able to determine when they are likely correct, or when they might need a second opinion. This is also related to the concept of self-monitoring, which involves one’s awareness of the limits of their ability in a specific moment (Eva & Regehr, 2011). Theoretically, feedback improves calibration because it can correct people’s self-monitoring assessments in specific instances, which serves to raises awareness of mismatches between one’s estimated performance and one’s actual performance (Hattie & Timperley, 2007; Rawson & Dunlosky, 2007). Receiving negative feedback will allow the clinician to identify which cognitive processes were faulty and will give an opportunity for re-calibration, so that the same mistake will not be made again. Positive feedback, on the other hand, will reinforce the use of processes that led to a successful outcome (Croskerry, 2000). When no feedback is received, this is often interpreted as positive feedback, which in turn can lead to miscalibration and errors. Accurate feedback is therefore crucial for improving performance.

A comprehensive review by Wisniewski et al. (2020) has shown that feedback is beneficial overall, but that specific forms of feedback are more effective. Feedback can broadly be divided in two types: performance feedback and information feedback. Performance feedback only informs the recipient of whether their response was correct or not, while information feedback not only helps one understand what mistake they made, but also why they made it and how they can avoid it in the future. Information feedback is generally found to be the more effective form of feedback (Archer, 2010; Hattie & Timperley, 2007; Wisniewski et al., 2020). Performance feedback in clinical practice can be as simple as determining if the patient’s treatment was successful or not; information feedback expands on that and could include additional information such as what treatment was ultimately successful or which follow-up tests or results provided further insights. Despite the fact that research specifically concerning calibration and feedback on the diagnostic process remains scarce, previous studies have shown that performance feedback could improve calibration on easy clinical cases (Nederhand et al., 2018) but not on difficult cases (Kuhn et al., 2022). It has been suggested that information feedback is needed to improve the diagnostic process (Archer, 2010; Ryan et al., 2020), though evidence for its effects remains limited (Kornegay et al., 2017).

Unfortunately, feedback for individual clinicians, especially regarding incorrect diagnoses, is rarely provided in clinical practice (Berner & Graber, 2008; Burgess et al., 2020; Schiff, 2008; Zwaan & Hautz, 2019), despite evidence that the confidence–accuracy calibration of clinicians is poor and gets worse as cases get more difficult (Meyer et al., 2013). Clinicians are often found to be overconfident (Friedman et al., 2005). This overconfidence is thought to be a part of human nature: people often underestimate the actual frequency of errors and even if they acknowledge errors occur, they often attribute them to others (Berner & Graber, 2008). Because clinicians rarely receive feedback on their diagnoses, they are not aware of their actual error rates and instead are implicitly led to believe they are often correct (Croskerry, 2000). Improving calibration via feedback could help clinicians in re-calibrating and improving their performance, which will in turn prevent diagnostic errors (Berner & Graber, 2008; Meyer & Singh, 2019; Schiff, 2008; Zwaan & Hautz, 2019).

This study examined the effect of performance feedback and information feedback on calibration and other aspects of the diagnostic process, compared to a control condition that did not receive feedback. The diagnostic process was measured in terms of diagnostic accuracy, confidence, calibration, and time to diagnose for medical interns diagnosing chest X-rays. We hypothesized that both performance feedback and information feedback would make students aware of their errors and would allow them to improve their calibration compared to the no feedback condition. We further expected that only information feedback would lead to a significant improvement in the diagnostic process. Information feedback teaches students to correct mistakes in their reasoning, whereas performance feedback would only make students more aware of their limitations without offering solutions (Ryan et al., 2020). We expected this to be reflected in the time to diagnose: information feedback might reduce time to diagnose in the test phase compared to the no feedback condition, because interns could use the feedback to become more proficient at correctly diagnosing X-rays. Conversely, we expected time to diagnose to increase in the performance feedback condition, as students would be aware that they have made mistakes and should spend more time thinking about the correct responses, but do not have the information to help them correct diagnoses efficiently.

We further explored confidence and calibration by comparing calibration between easier and more difficult cases. Prior research has shown poorer calibration for more difficult cases (Meyer et al., 2013). With a wider gap between accuracy and confidence, we were interested in exploring whether feedback would have an even larger impact on difficult cases relative to easier cases.

## Methods

### Ethics approval

The study was approved by the medical ethical committee of the Erasmus University Medical Center (Erasmus MC) (MEC-2021-0808). All participants gave informed consent. All methods were carried out in accordance with the relevant guidelines and regulations.

### Design

**Fig. 1:**
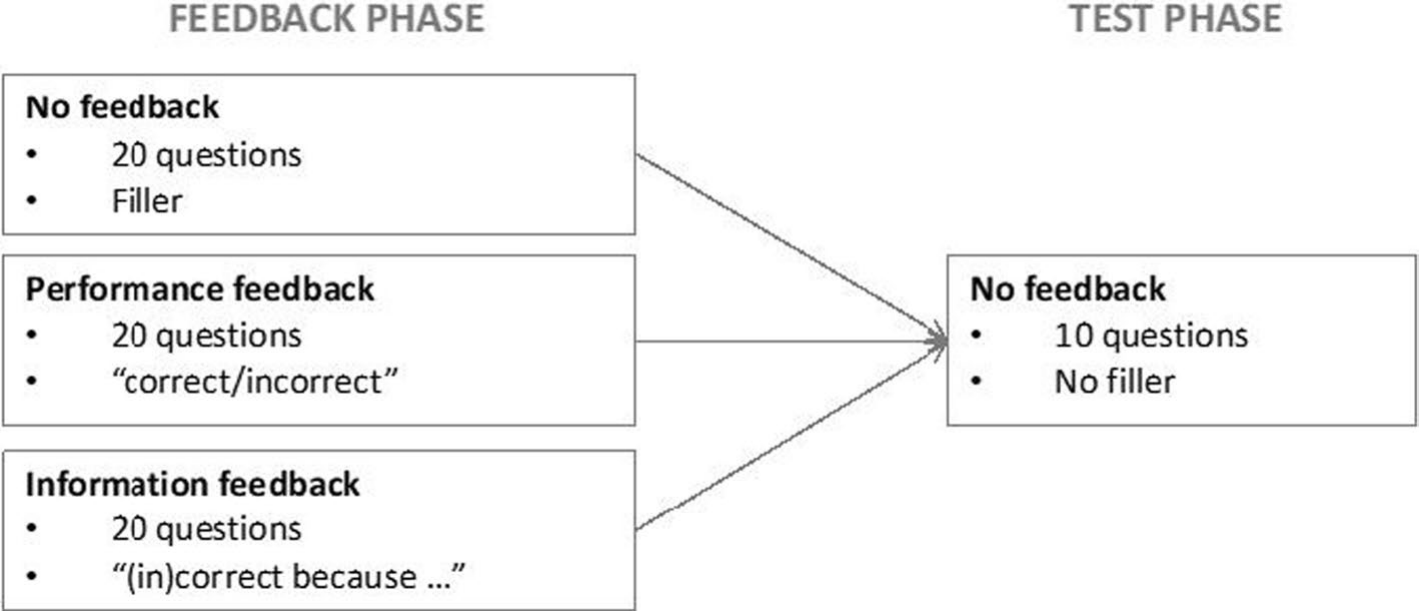
Study design

We conducted a computer-based experiment with a 2 (phase) × 3 (feedback condition) mixed design. Participants completed the feedback phase first, followed by a test phase. In the feedback phase, participants were randomly divided into one of three conditions (no feedback, performance feedback, or information feedback) and diagnosed 20 chest X-rays (Fig. 1). After participants entered a diagnosis, they were shown the same image again. Those in the no feedback condition received no extra information on their diagnosis or the X-ray itself. Those in the performance feedback condition were shown whether their diagnosis was correct or incorrect. Finally, participants in the information feedback condition received the correct diagnosis, with the addition of an explanation on how the correct diagnosis could be identified (“Appendix 1”). Time to view the X-ray or feedback was not restricted. In the test phase, participants diagnosed 10 new X-rays without receiving feedback.

### Participants

Interns in at least their fourth year of Dutch medical school, who were about to start clinical internships, were recruited during class, through online student portals, and via social media. The estimated sample size was calculated using G-power 3.1.9.7 (Faul et al., 2007) for one-way analysis of variance (ANOVA) with a power of 0.80, α of 0.05, and a medium effect size of 0.3 based on Nederhand et al. (2018) This resulted in an estimated sample size of 111 participants.

### Materials

Thirty chest X-rays representing five diagnoses (i.e., atelectasis, pleural effusion, pneumothorax, tumor, or no abnormality) were selected from the Erasmus MC database and external open access databases. The diagnoses were confirmed by CT scans. Per diagnosis, four X-rays were selected for the feedback phase and two for the test phase. Cases were matched across phases on diagnosis and difficulty level, ensuring that the cases were comparable. The difficulty level was judged for the level of medical interns with little experience and confirmed by an internist (JA), a medical doctor (RB), and a final year medical student (MS). The cases were classified as easy if all three experts could diagnose the X-ray correctly and as difficult if only two of the three experts could diagnose the X-ray correctly. This was performed to ensure a balanced set of easy and difficult cases was used.

### Procedure

The experiment was conducted using an online questionnaire prepared in Qualtrics (an online survey tool). Upon starting the experiment, participants received an information letter and were asked to sign informed consent. They were fully informed about the goal of the study. Participants then filled out general demographics (i.e., age, sex, attended university, years studying medicine, and attended clerkships). During the feedback phase, participants were randomized into one of the three feedback conditions. For each case, they had to select the most likely diagnosis out of five possible diagnoses from a drop-down menu and then were asked to indicate how confident they were in this diagnosis. Then, in the test phase, participants diagnosed ten new chest X-rays without feedback and marked their confidence per case. After completing the experiment, all participants received information feedback on the test phase X-rays and in addition, the no feedback condition received information feedback on the feedback phase X-rays (“Appendix 1”).

### Outcome measures

The independent variable was the type of feedback participants received in the feedback phase. This was no feedback (control condition), performance feedback, or information feedback. The dependent variables were diagnostic accuracy, confidence, confidence–accuracy calibration, and time to diagnose. For diagnostic accuracy, selection of the correct diagnosis was scored as 1, any other answer was scored as 0, based on pre-established diagnoses. We further measured confidence on a scale from 0 to 10, from “very not confident” to “very confident”. Confidence–accuracy calibration was derived from the diagnostic accuracy and confidence measures. Finally, time to diagnose was measured in seconds from the moment participants began diagnosing a case until they submitted a diagnosis.

### Statistical analysis

Diagnostic accuracy, confidence, confidence–accuracy calibration, and time to diagnose were assessed using one-way ANOVAs as a function of feedback type. In cases where data were not normally distributed, we performed a Kruskal–Wallis test (non-parametric ANOVA) instead. All reported post hoc tests were corrected using the Bonferroni method. We focused on the results from the test phase because the intervention needed to be finished before its effects could be measured. We assumed significance if *p* < 0.05. All tests were performed in IBM SPSS Statistics (Version 28, Armonk, NY: IBM Corp).

Confidence–accuracy calibration was derived by plotting the mean diagnostic accuracy and mean confidence for each condition. For this, the mean accuracy was converted into a percentage and the mean confidence was multiplied by ten to make it comparable to accuracy. Calibration was additionally quantified using the R^2^ as a measure of goodness-to-fit to a scatterplot of the mean confidence and mean accuracy per condition. This was done according to the method described by Staal et al. (2021) in which a higher R^2^-value indicated a better calibration.

Furthermore, we performed one pre-planned and one post-hoc exploratory analysis to further investigate confidence and calibration. In the pre-planned comparison, we compared the effects of feedback on diagnostic accuracy, confidence, calibration, and time to diagnose separately for easy and difficult cases using a paired *t *test. In the post-hoc analysis, we compared average confidence over all test phase cases for the 25% worst and 25% best performing students and compared the outcomes using a between subjects *t *test.

## Results

### Demographics

A total of 125 medical interns volunteered and 116 completed both the feedback and the test phases. 45 participants were randomized into the no feedback condition, 38 into the performance feedback condition, and 42 into the information feedback condition. Participant demographics are displayed in Table 1. Means of all outcome measures for the three feedback conditions are listed in Table 2.

**Table 1:** Participant demographics. A total of 125 interns participated

Age [mean (SD)]	Sex [N (%) female]	University [N (%) Erasmus MC]	Time studying medicine [mean (SD)]	Attended clinical clerkships [N (%)]		
				None	Internal medicine	Multiple
23 (2) years	93 (74.4%)	118 (94.4%)	53 (21) months	51 (40.8%)	53 (42.4%)	21 (16.8%)

**Table 2:** Overview of means and 95% CI for performance in the test phase, per feedback condition

	Condition					
	No feedback		Performance feedback		Information feedback	
	Mean (SD)	95% CI	Mean (SD)	95% CI	Mean (SD)	95% CI
*Outcome measure*						
Diagnostic accuracy (0–1)	0.49 (0.2)	[0.43–0.55]	0.65 (0.2)	[0.58–0.72]	0.68 (0.2)	[0.60–0.75]
Confidence (0–10)	5.74 (1.1)	[5.40–6.09]	6.38 (1.6)	[5.82–6.93]	6.39 (1.2)	[6.02–6.75]
Time to diagnose (in s)	16.98 (7.2)	[14.70–19.27]	15.02 (5.0)	[13.27–16.78]	19.13 (16.1)	[14.06–24.20]

### Main analyses

Data for diagnostic accuracy and time taken to diagnose were not normally distributed, so we performed a Kruskal–Wallis test.

#### Diagnostic accuracy

Diagnostic accuracy between feedback conditions differed significantly overall (*F*(2) = 18.06, *p* < 0.001). Post-hoc analysis showed that the no feedback condition scored lower than the performance feedback condition (*F*(2) = − 25.25, *p* = 0.003, *d* = 0.79) and the information feedback condition (*F*(2) = − 29.02, *p* < 0.001, *d* = 0.86). The feedback conditions did not differ significantly (*F*(2) = − 3.78, *p* = 1.000).

#### Confidence

Overall, confidence differed significantly between all feedback conditions (*F*(2) = 3.29, *p* = 0.041); however, no significant differences were found in the pairwise post-hoc comparisons between the conditions (*p* > 0.050 for all).

#### Confidence–accuracy calibration

**Fig. 2:**
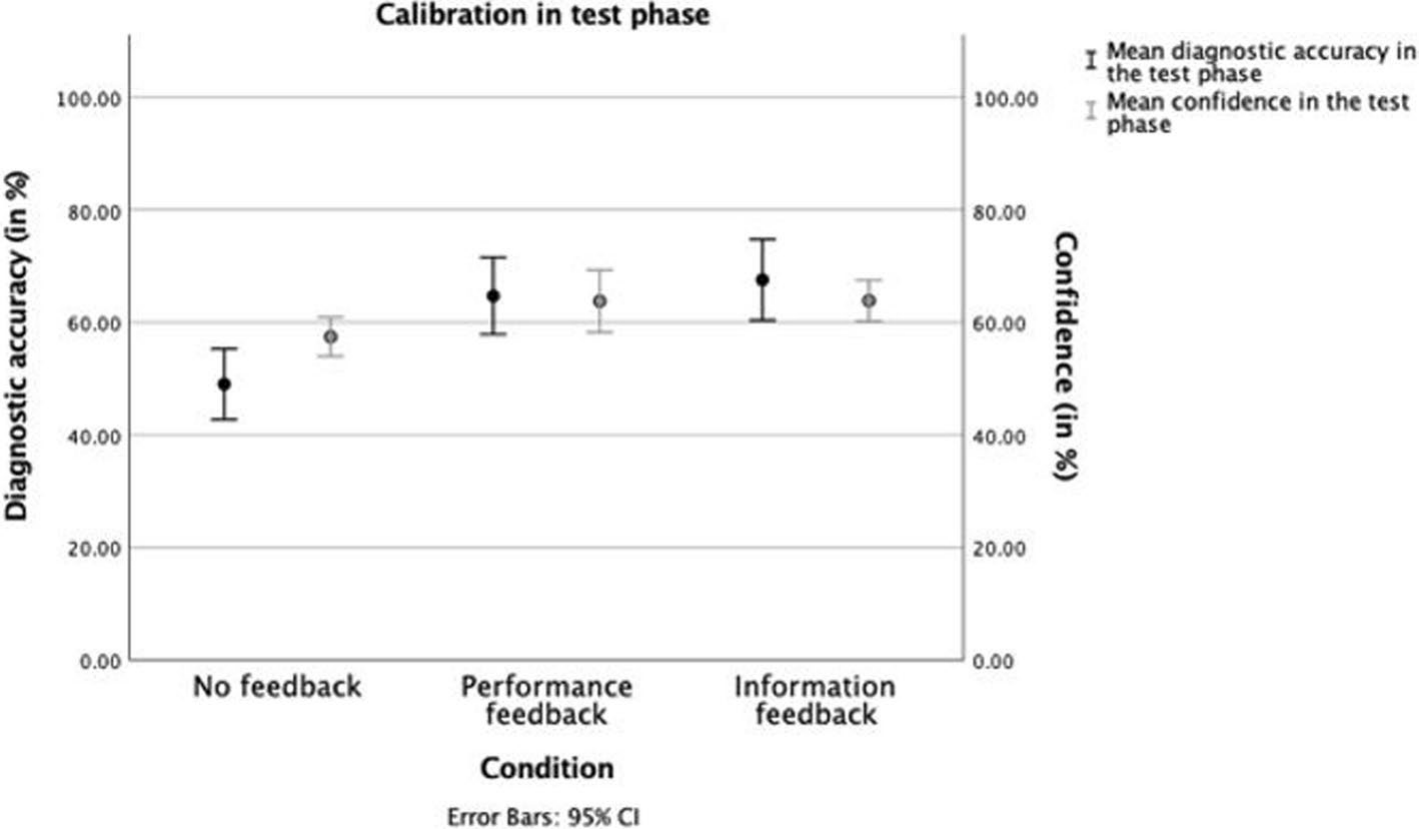
Mean accuracy and confidence results of the test phase per feedback condition. Error bars represent the 95% CI

We now present the main variable of interest, which is derived from the preceding data on accuracy and confidence. Mean diagnostic accuracy was overall well-aligned with mean confidence (Fig. 2). The confidence–accuracy calibration was lowest in the no feedback condition (R^2^ = 0.05). Both feedback conditions achieved better calibration, with information feedback showing the highest calibration (performance feedback: R^2^ = 0.12; information feedback: R^2^ = 0.19) (“Appendix 2”).

#### Time to diagnose

Between the three conditions, there were no significant differences in time spent on diagnosing the cases (*F* (2) = 3.24, *p* = 0.197).

### Exploratory analyses

As mentioned in the introduction, exploratory analyses were performed to further understand our results and the impact of feedback.

**Fig. 3:**
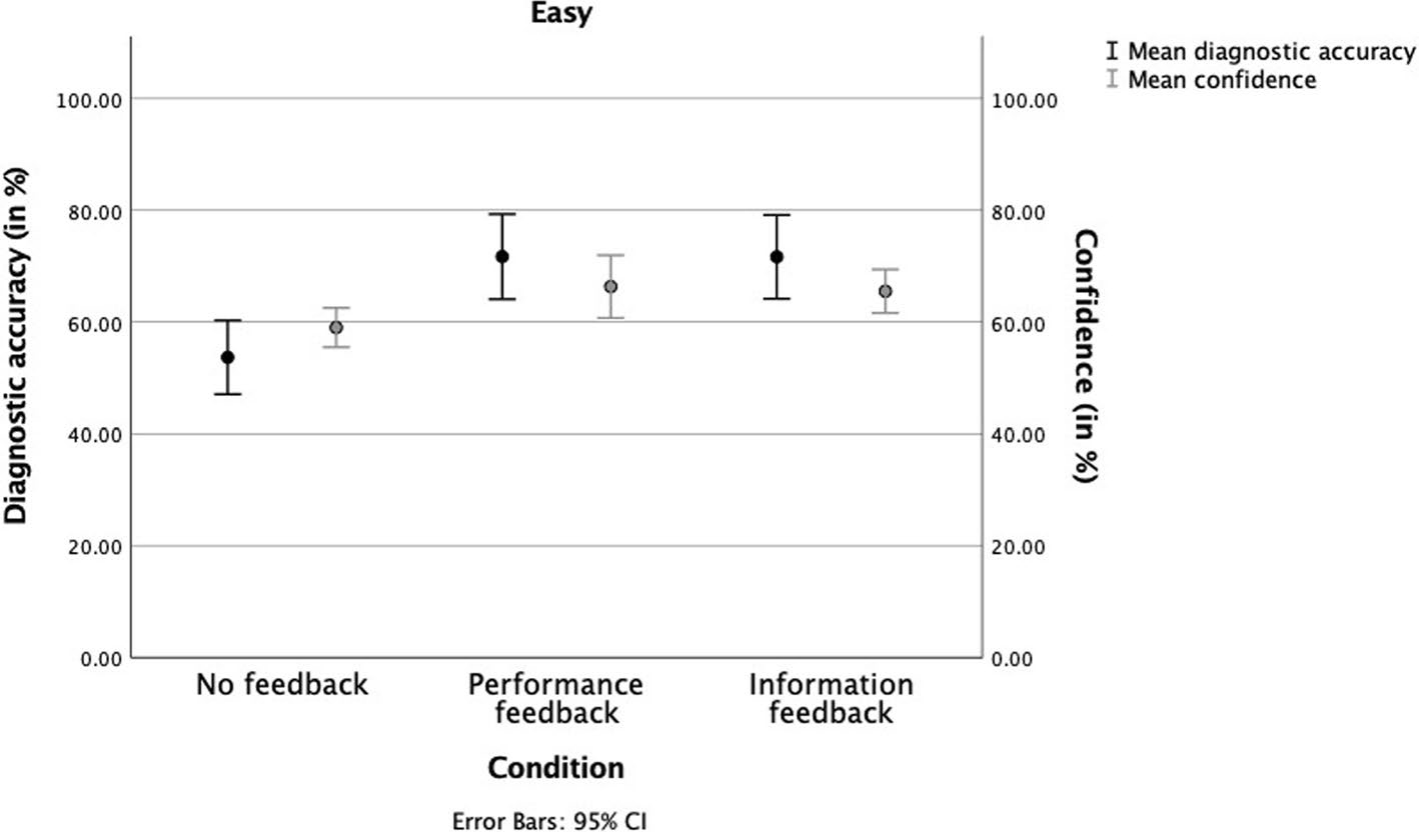
Interns’ mean diagnostic accuracy and confidence scores per feedback condition for easy cases. Error bars represent the 95% CI

**Fig. 4:**
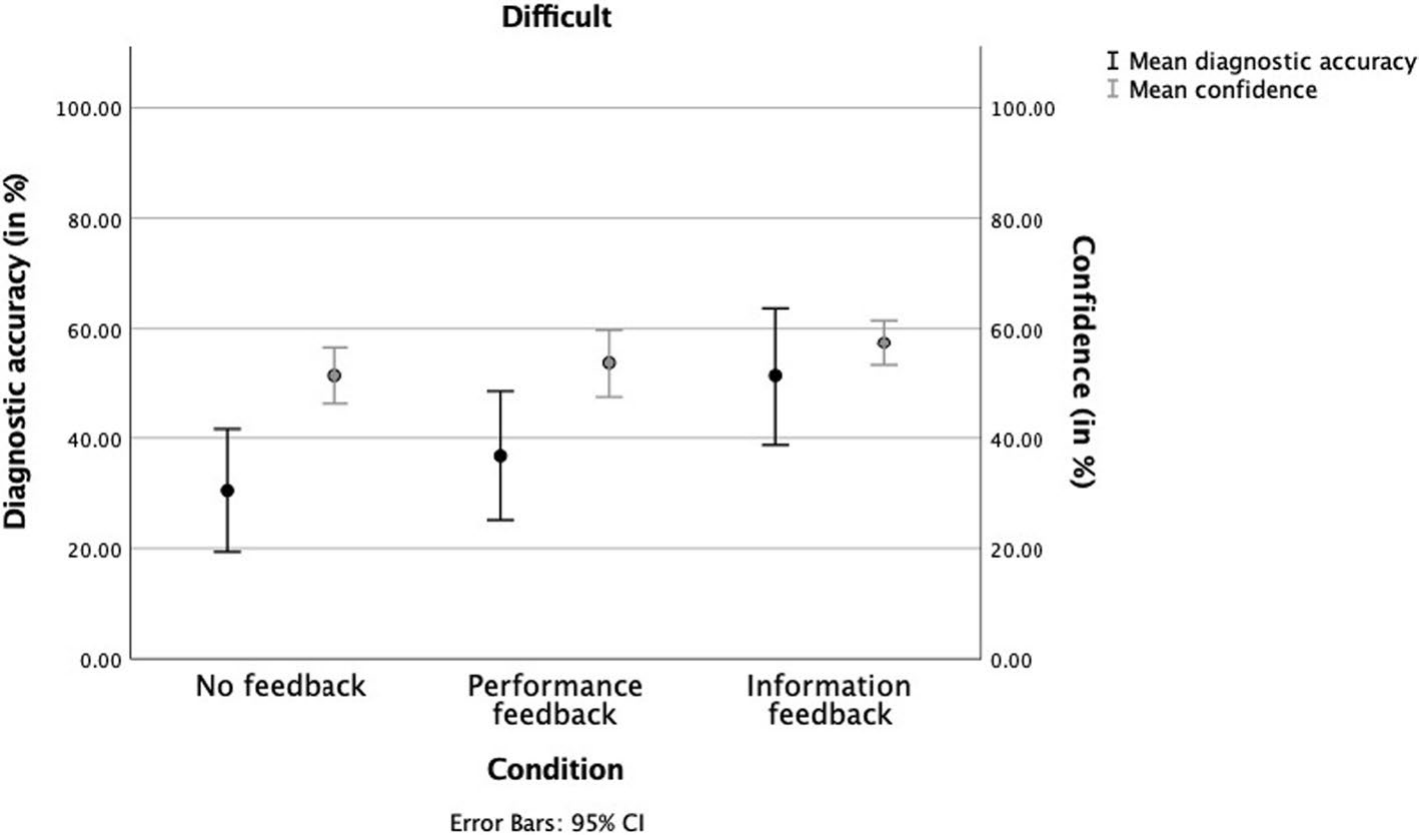
Interns’ mean diagnostic accuracy and confidence scores per feedback condition for difficult cases. Error bars represent the 95% CI

First, we plotted the results separately for easy and difficult cases (see Figs. 3, 4). Overall, mean diagnostic accuracy was significantly lower (*t*(115) = 7.37, *p* < 0.001) for difficult cases (*M* = 0.40, *SD* = 0.37) compared to easy cases (*M* = 0.65, *SD* = 0.24). The same was true for mean confidence (*t*(115) = 8.17, *p* < 0.001) for difficult (*M* = 5.41, *SD* = 1.57) compared to easy cases (*M* = 6.34, *SD* = 1.35).Confidence–accuracy calibration was better for easy cases (R^2^ = 0.18) (Fig. 3), compared to difficult cases (R^2^ = 0.02) (Fig. 4). The calibration for easy cases was worst in the no feedback condition (R^2^ = 0.06) and improved in the feedback conditions, with information feedback showing the highest calibration (performance feedback: R^2^ = 0.11, information feedback: R^2^ = 0.22). Feedback did not improve calibration in difficult cases (no feedback: R^2^ = 0.01, performance feedback: R^2^ = 0.02, information feedback: R^2^ = 0.01).

Second, we selected the 25% lowest scoring interns (*N* = 32, average test phase accuracy ≤ 0.4) and the 25% highest scoring interns (*N* = 39, average test phase accuracy ≥ 0.8). Among the 32 lowest scoring interns, 19 had been assigned to the no feedback condition, 7 to the performance feedback condition, and 6 to the information feedback condition. Among the 39 highest scoring interns, 6 had been assigned to the no feedback condition, 14 to the performance feedback condition, and 19 to the information feedback condition.

Confidence for the lowest scoring interns was not normally distributed (*p* = 0.042), though it was normally distributed for the highest scoring interns (*p* = 0.200). Given that a non-parametric test gave the same results as the *t* test, we reported the *t* test. The 25% best performing interns were more confident (*M* = 6.8, *SD* = 1.26) than the 25% worst performing interns (*M* = 5.4, *SD* = 1.34; *p* < 0.001). The best performing interns were underconfident whereas the worst performing interns were overconfident about their performance.

## Discussion

The current study examined the impact of performance feedback and information feedback, compared to a control condition who did not receive feedback, on the confidence–accuracy calibration and diagnostic process of medical interns who diagnosed chest X-rays. Both types of feedback improved diagnostic accuracy. Confidence increased in both feedback conditions; this increase especially stands out compared to the small confidence intervals around interns’ average reported confidence. Although the difference was no longer significant in the post-hoc tests, it indicates that confidence was influenced by feedback. In line with our hypothesis, overall calibration improved in both feedback conditions as compared to the no feedback condition. Contrary to our hypothesis, time to diagnose did not differ between the conditions.

Further exploratory analyses indicated that interns’ confidence seemed at least somewhat sensitive to their performance, as the 25% worst performing interns reported lower confidence than the 25% best performing interns and confidence was lower for more difficult cases. However, we cannot be sure of the underlying mechanisms and should keep in mind that people often show a tendency to score more towards the middle of a scale (to 50% confidence in this case), which would also result in the pattern we observe. For easy cases, interns were overall well-calibrated and calibration increased in the feedback conditions; for difficult cases calibration was poor and was not affected by feedback condition, though future research should replicate these results in a larger sample of cases as the difficult case sample only consisted of two cases.

Our results regarding the positive impact of performance feedback on diagnostic accuracy and overall calibration are in line with previous studies (Dunlosky & Rawson, 2012; Lichtenstein & Fischhoff, 1980; Nederhand et al., 2018). We found good calibration in easy cases, similarly to Nederhand et al. (2018), along with an increase in calibration in the feedback conditions. In line with Kuhn et al. (2022), we also observed poorer calibration in difficult cases, but we did not replicate their observation that participants became underconfident. If anything, participants in our study appeared to be more overconfident as opposed to underconfident. The positive effects of information feedback on the diagnostic process we observed are in line with previous work, though this work was not specifically aimed at medical education (Hattie & Timperley, 2007; Wisniewski et al., 2020). Lastly, we observed that performance feedback and information feedback were equally effective, contrary to Ryan et al. (2020), who proposed that information feedback was superior as it has the potential to fill knowledge gaps.

Although our study indicated that feedback was overall beneficial to calibration, it remains difficult to determine what processes underlie this improvement. One possible explanation is that calibration improved as a result of interns’ improved accuracy rather than a change in their confidence. We observed a similar pattern as Meyer et al. (2013) who showed that clinician’s confidence was less sensitive to changes in their accuracy, as confidence was relatively stable across easy and difficult cases despite larger fluctuations in accuracy. On the other hand, our exploratory analyses suggested that interns’ were at least somewhat sensitive to case difficulty, as confidence was significantly lower for the 25% worst performing interns compared to the 25% best performing interns, and confidence was lower for difficult cases relative to easy cases. Further research is necessary to understand what exactly we are measuring when we ask clinicians for their subjective confidence: perhaps confidence also reflects clinicians’ decision threshold, or how certain they want to be before they decide on a diagnosis. In that case, the measure would be expected to remain stable. It will be crucial to understand clinician’s confidence and how we measure it before we can improve calibration.

In summary, the current study shows that clinicians’ calibration can be improved by feedback. However, this improvement was mostly limited to easier cases, suggesting that another approach will likely be needed to improve calibration in difficult cases. Feedback relies on the ability of the learner to recognize and improve on their mistakes, which is difficult to achieve in tasks that have a high complexity for the learner (Kluger & DeNisi, 1996). If implemented over the course of an entire curriculum, however, learners might gain more insight in their general performance and might become more effective learners over time. After all, as they are taught more, less material will be too complex and more material will become easier, which would also increase the impact of feedback. This approach might be specifically suitable to education involving progress tests and other assessments that allow improvement over time (Wrigley et al., 2012). Overall, feedback remains a valuable intervention, given its effectiveness in improving diagnostic accuracy without significantly increasing time spent to diagnose. The latter might be attributed to our use of chest X-rays, as visual cases are usually diagnosed quicker. Furthermore, suggestions to give feedback on the diagnostic process of clinicians are becoming more frequent and our findings support this endeavor (Schiff, 2008). There are ideas to standardize communicating the final diagnosis of a patient to the clinician who had seen the patient (Branson et al., 2021; Lavoie et al., 2009; Shenvi et al., 2018). Future research should replicate the current findings in more experienced clinicians and test the implementation of both feedback types in practice.

This study has several strengths and limitations. Strengths include the experimental design with control condition, ensuring that effects seen in the between subjects analyses could be distinguished from learning effects between the two phases. Furthermore, all included chest X-rays had confirmed diagnoses and we could include a large number of cases because we used visual cases. This is important because sufficient practice is necessary to see effects of feedback. Limitations include that we only tested medical interns on visual images, meaning that the results are not generalizable to other levels of expertise, other types of cases, or to practice. Further, the test phase occurred immediately after the feedback phase. A time gap would have allowed participants more time to incorporate the intervention in their learning and might have a larger effect in the test phase (Mamede et al., 2012). Another limitation was the multiple choice format for diagnosis: participants could have selected the correct diagnosis per exclusionem. However, providing too many options (i.e., via free text response) could have overwhelmed our relatively inexperienced participants. Future research should investigate if the effects of feedback remain when these factors are accounted for.

In conclusion, clinicians’ confidence–accuracy calibration could be improved with both performance and information feedback, though exploratory results indicate this was limited to easier cases. More research will be needed to understand the relationship between feedback and calibration, however, for example by replicating these results in other, non-visual specialties, and in more experienced participants. Overall, feedback is a promising intervention that has the potential to improve both clinicians’ actual diagnostic accuracy and their estimation of their own accuracy in cases that are not too complex for the learner, as well as the potential to reduce diagnostic errors.

## Figures and Tables

**Fig. 5:**
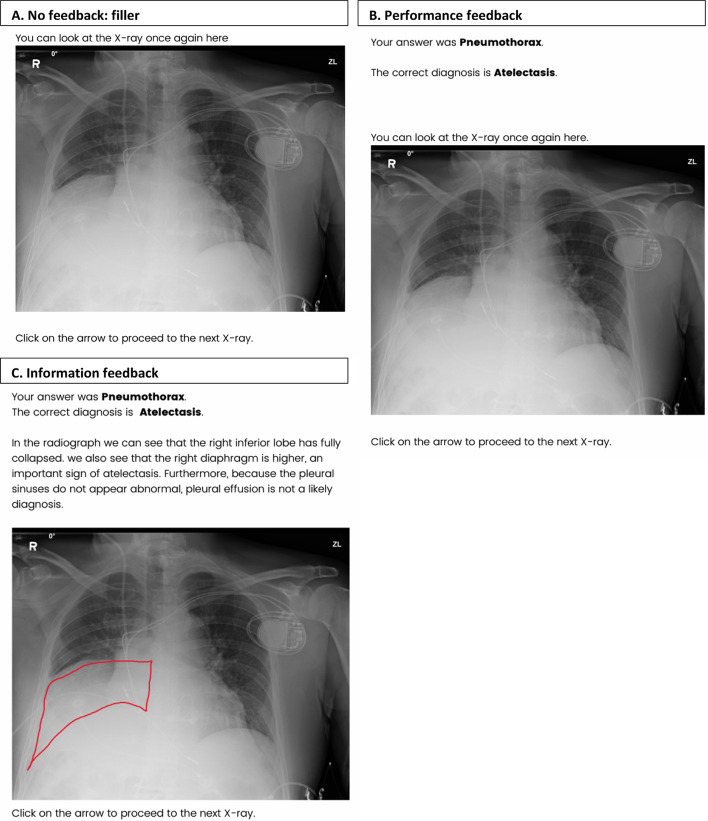
**a** Filler task in the control condition, **b** performance feedback, **c** information feedback. After the experiment, all participants received information feedback (**c**) on cases they had not previously received feedback for

**Fig. 6:**
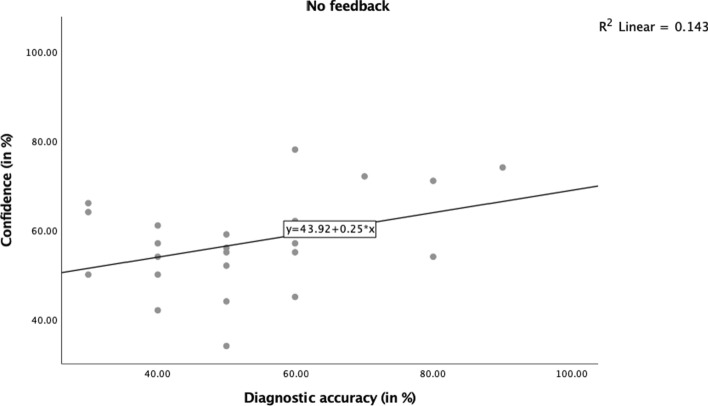
Scatterplot of the mean confidence and mean diagnostic accuracy of each participant in the no feedback condition.

**Fig. 7:**
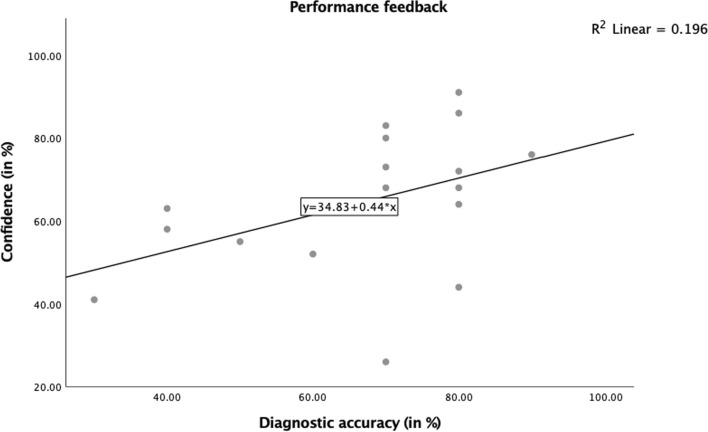
Scatterplot of the mean confidence and mean diagnostic accuracy of each participant in the performance feedback condition

**Fig. 8:**
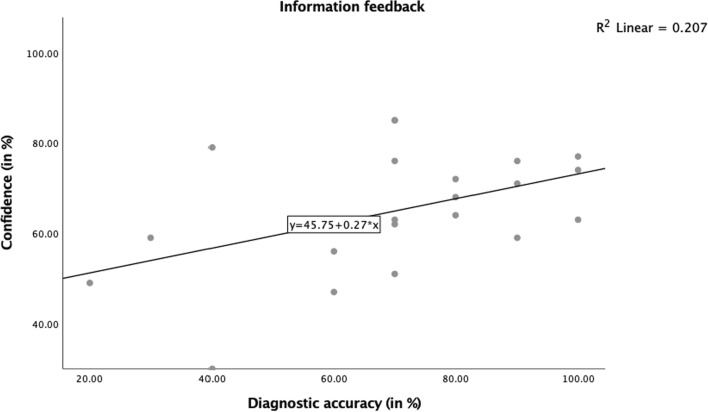
Scatterplot of the mean confidence and mean diagnostic accuracy of each participant in the information feedback condition

## Data Availability

The study protocol was preregistered and is available online at Open Science Framework (https://osf.io/tvw8e).

## Appendix 1: Feedback conditions

Figure 5 shows an example of the feedback and fillers participants received in each condition.

## Appendix 2: Calibration

Scatterplots of the relationship between mean accuracy and mean confidence over all cases (no feedback group: Fig. 6; performance feedback group: Fig. 7; information feedback group: Fig. 8). The R^2^ is a measure for calibration, which is expresses how well the data fit a linear model.   

## Abbreviations

ANOVA: Analysis of variance
Erasmus MC: Erasmus University Medical Center
